# Complete Genome Sequence of Chikungunya Virus Isolated in the Philippines

**DOI:** 10.1128/genomeA.00336-14

**Published:** 2014-06-26

**Authors:** Kent D. Kawashima, Lady-Anne C. Suarez, Hannah Karen M. Labayo, Veni R. Liles, Noel C. Salvoza, David C. Klinzing, Maria Luisa G. Daroy, Ronald R. Matias, Filipinas F. Natividad

**Affiliations:** Research and Biotechnology, St. Luke’s Medical Center, Quezon City, Philippines

## Abstract

Chikungunya virus is an alphavirus of the *Togaviridae* family, which causes a febrile illness with arthralgia in humans. We report here on the complete genome sequence of chikungunya virus strain CHIKV-13-112A isolated from a patient in the Philippines who was suspected to have dengue virus. Phylogenetic analysis revealed that the strain is of the Asian genotype.

## GENOME ANNOUNCEMENT

Chikungunya virus (CHIKV) is an alphavirus that causes chikungunya fever. It is transmitted by the bite of an infected *Aedes* mosquito. CHIKV occurs throughout the Philippines, with outbreaks reported in many provinces in 2012 to 2013. (see http://www.promedmail.org/direct.php?id=1494945 and http://www.promedmail.org/direct.php?id=1980717).

Here, we report the complete coding sequence of the Asian genotype strain CHIKV-13-112A isolated from a Filipino patient initially suspected to have dengue. CHIKV was initially detected by reverse transcription-PCR (RT-PCR) targeting the E1 gene (1) and propagated in C6/36 *Aedes albopictus* cells for 7 days. CHIKV RNA isolated from the culture supernatant was used to construct a tagged random cDNA library by a modified sequence-independent single-primer amplification (SISPA) method (2). First-strand tagged cDNA was produced using SuperScript III reverse transcriptase (Invitrogen) and the second-strand cDNA using the Klenow fragment (Vivantis). Tagged double-stranded (ds)-cDNA was amplified using iProof high-fidelity (HF) Taq polymerase (Bio-Rad) and a 20-nucleotide (nt) primer, FR20RV, which is complementary to the palindromic tag on ds-cDNA. Amplified ds-cDNA was used for library preparation for next-generation sequencing on MiSeq (Illumina). Paired-end sequencing was carried out using MiSeq version 2 reagents. The reads were assembled *de novo* using SGA (version 0.10.13) (3) and mapped to the CHIKV complete genome sequence most similar to the assembled contigs (accession no. KF318729) using the Burrows-Wheeler aligner (BWA) (version 0.7.8) (4). The consensus sequence of the mapped reads was determined using SAMtools (version 0.1.19-44428cd) (5).

The CHIKV-13-112A genome obtained is a positive single-stranded RNA (ssRNA) that is 11,933 nt long. Its coding sequence consists of two open reading frames (ORFs) flanked by 5′ and 3′ untranslated regions (UTR) whose termini were not verified. The first 7,413-nt ORF encodes a 2,471-amino acid (aa) nonstructural polyprotein containing nonstructural (ns)P1, 2, 3, and 4 proteins, respectively. There is a read-through site at positions 5586 to 5588 within the ORF that ignores an internal stop codon. From positions 7438 to 7502, there is a 65-nt UTR separating the two ORFs. The second 3,747-nt ORF encodes a 1,249-aa structural polyprotein containing the C, E2, E3, and E1 proteins, respectively.

The CHIKV-13-112A and other CHIKV complete genome sequences in GenBank, together with those of O’nyong-nyong virus (accession no. NC_001512), were aligned using MUSCLE (version 3.8.31) (6) and manually adjusted in UGENE (version 1.11.5) (7). Phylogenetic tree reconstruction based on the complete coding sequences was performed through RAxML (version 7.2.8) (8) using the GTRGAMMA model of rate heterogeneity. The results revealed that strain CHIKV-13-112A belongs to the Asian genotype. A BLASTn search revealed 99% similarity to an isolate from Zhejiang, China (accession no. KF318729), while a phylogenetic analysis of complete coding sequences showed close relation to another Chinese strain (accession no. KC488650) in addition to the Zhejiang isolate, and to an Indonesian strain (accession no. FJ807897), all of which form a subclade in the Asian genotype. Likewise, two other CHIKV Philippine strains isolated in 1985 were classified as belonging to the Asian genotype (9). This confirms our previous analyses based on the nsP1 and E1 genes of 12 CHIKV strains isolated in 2012 from Manila, Philippines, which showed them to be of the Asian genotype (K. Kawashima, L. Suarez, H. Labayo, N. Salvoza, M. Daroy, R. Matias, and F. Natividad, unpublished data).

### Nucleotide sequence accession numbers.

The complete genome of CHIKV-13-112A has been deposited in DDBJ with the accession no. AB860301. The version described in this paper is the third version, AB860301.3.
